# Medieval mummies of Zeleny Yar burial ground in the Arctic Zone of Western Siberia

**DOI:** 10.1371/journal.pone.0210718

**Published:** 2019-01-25

**Authors:** Sergey Mikhailovich Slepchenko, Alexander Vasilyevich Gusev, Evgenia Olegovna Svyatova, Jong Ha Hong, Chang Seok Oh, Do Seon Lim, Dong Hoon Shin

**Affiliations:** 1 Tyumen Scientific Center of the Siberian Branch of the Russian Academy of Sciences, Tyumen, Russia; 2 Arctic Research Center, Archeology Department, Archeology and Ethnology Sector, Salekhard, Russia; 3 Institution of Culture of Sverdlovsk Region, Center for Protection and Use of Monuments of History and Culture of Sverdlovsk Region, Scientific and Production Center, Ekaterinburg, Russia; 4 Lab of Bioanthropology, Paleopathology, and History of Diseases, Department of Anatomy/Institute of Forensic Science, Seoul National University College of Medicine, Seoul, South Korea; 5 Department of Dental Hygiene, College of Health Science, Eulji University, Seongnam, South Korea; Hebrew University, ISRAEL

## Abstract

Notwithstanding the pioneering achievements of studies on arctic mummies in Siberia, there are insufficient data for any comprehensive understanding of the bio-cultural details of medieval people living in the region. In the Western Siberian arctic, permafrost mummies have been found in 12^th^ to 13^th^ century graves located in the Zeleny Yar (Z-Y) burial ground (66°19'4.54"С; 67°21'13.54"В). In 2013–2016, we were fortunate to be able to excavate that cemetery, locating a total of 47 burials, including cases of mummification. Some of these mummies had been wrapped in a multi-layered birch-bark cocoon. After removal of the cocoon, we conducted interdisciplinary studies using various scientific techniques. Gross anatomical examination and CT radiography showed that the internal organs were still well preserved inside the body cavities. Under light and electron microscopy, the histological findings were very similar to those for naturally mummified specimens discovered in other countries. Ancient DNA analysis showed that the Z-Y mummies’ mtDNA haplotypes belong to five different haplogroups, namely U5a (#34), H3ao (#53), D (#67–1), U4b1b1 (#67–2), and D4j8 (#68), which distinguish them for their unique combination of Western- and Eastern Siberia-specific mtDNA haplogroups. Our interdisciplinary study obtained fundamental information that will form the foundation of successful future investigations on medieval mummies found in the Western Siberian arctic.

## Introduction

Over the course of the past several decades, different kinds of mummies have been discovered, attracting the attention of scholars around the world [1–3]. As for the question of how the remains had become mummified, there are two main hypotheses proposed thus far: natural and artificial mummification [1]. Natural mummification typically occurs either in extremely dry or permafrost conditions [3]. For instance, in polar-climatic zones or high-altitude mountainous areas, bodies are often mummified by the combination of high wind, low temperature, and humidity [4,5]. By repeated freezing or evaporation, water continues to be removed from the tissues; the body’s complete putrefaction and decomposition thus prevented, it finally becomes mummified [4].

For several decades, various research groups have carried out interdisciplinary investigations on permafrost mummies discovered in Alaska, Canada, and Greenland [4]. Although not widely known to the outside world, permafrost mummies found in the Russian Federation have been reported as well. For example, mummies were found in a high-altitude area of the Ukok plateau of the Altay Mountains [6]. Additionally, well-preserved mummies have been discovered in medieval Siberian cemeteries. Permafrost mummies found in burial grounds of the Sakha Republic (Yakutiya) provided a rare opportunity to conduct a detailed investigation of historical native East Siberian peoples [7–9].

Certainly, permafrost mummies discovered in Russia (or elsewhere), with their intact organs and tissues, have great academic value for their utility in revealing the health and disease status of medieval peoples. Nonetheless, Siberia being an immensely vast area, reports therefrom are still insufficient for any complete reconstruction of the cultures of historical Siberian peoples. Especially for the areas west of the Yenisei River (Western Siberia), the research remains scanty, notwithstanding the many ancient communities that have existed and prospered there.

In this regard, a relatively recent series of archaeological excavations undertaken in the arctic permafrost area of Yamalo-Nenets Autonomous Okrug (YaNAO) in Western Siberia are highly significant. Between 1999 and 2002, under the leadership of N.V. Fedorova, archaeological investigations were conducted at the Zeleny Yar (Z-Y) site, during which, well-preserved mummies were discovered in graves [10,11]. The scientific analyses performed on those specimens [10–12] yielded very revealing anthropological, genetic and paleoparasitological information.

Despite these pioneering achievements, we must admit that the previous studies in their collectivity cannot shed light on any medieval Siberian people as a whole. In this regard, our interdisciplinary research team comprised of experts from the disciplines of archaeology, gross morphology, radiology, histology, and molecular biology recently performed concerted analyses on several mummies discovered at a total of 47 burial sites excavated by our archaeologist representatives at the Z-Y cemetery (2013–2016). The current report can be considered to be an important example of Z-Y mummies’ academic potential to reveal biocultural details of ancient native people who had inhabited the Western Siberian arctic.

## Archaeological considerations

**Fig 1:**
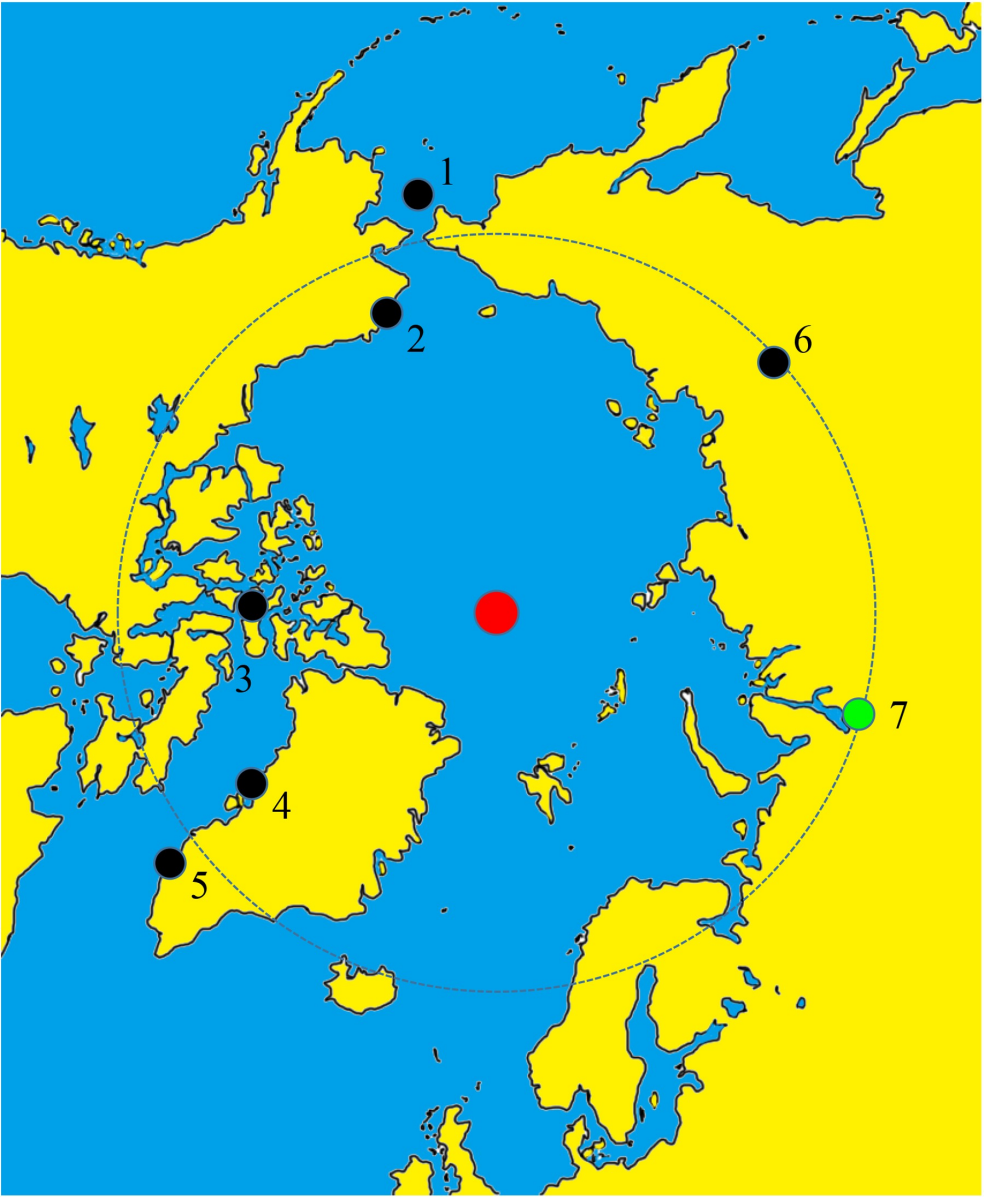
The Z-Y archaeological site (green dot) situated in Salekhard, Yamalo-Nenets Autonomous Okrug, Russian Federation. Other arctic or subarctic sites where permafrost mummies were discovered are marked in black dots: 1, St. Lawrence Is.; 2, Utqiagvik; 3, Beechey Is.; 4, Qilakitsoq; 5, Nuuk/Godthaalb; 6, Verhoyansk; 7, Salekhard. Red dot, the North Pole.

The Z-Y archaeological site (66°19'4.54"С; 67°21'13.54"В) is situated on a flood plain island that is located about 40 km from the city of Salekhard, YaNAO, Russian Federation (Fig 1). The cemetery was dated to about the 12^th^ to 13^th^ centuries based on the archaeological findings and dendrochronology data. As mentioned above, the cemetery was first investigated from 1999 to 2002. After a long break, a new excavation (2013–2016) at the Z-Y site began in 2013, thanks to a permit (Approval number: 422) issued by the YaNAO government for fieldwork at the identified archaeological heritage site known as “Zeleny-Yar Yard”. Archaeological investigations were performed in accordance with the related laws and bylaws currently enforced in the Russian Federation. We declare that there are no ethical conflicts with respect to our study’s potential impact on the people currently living in the area.

**Fig 2:**
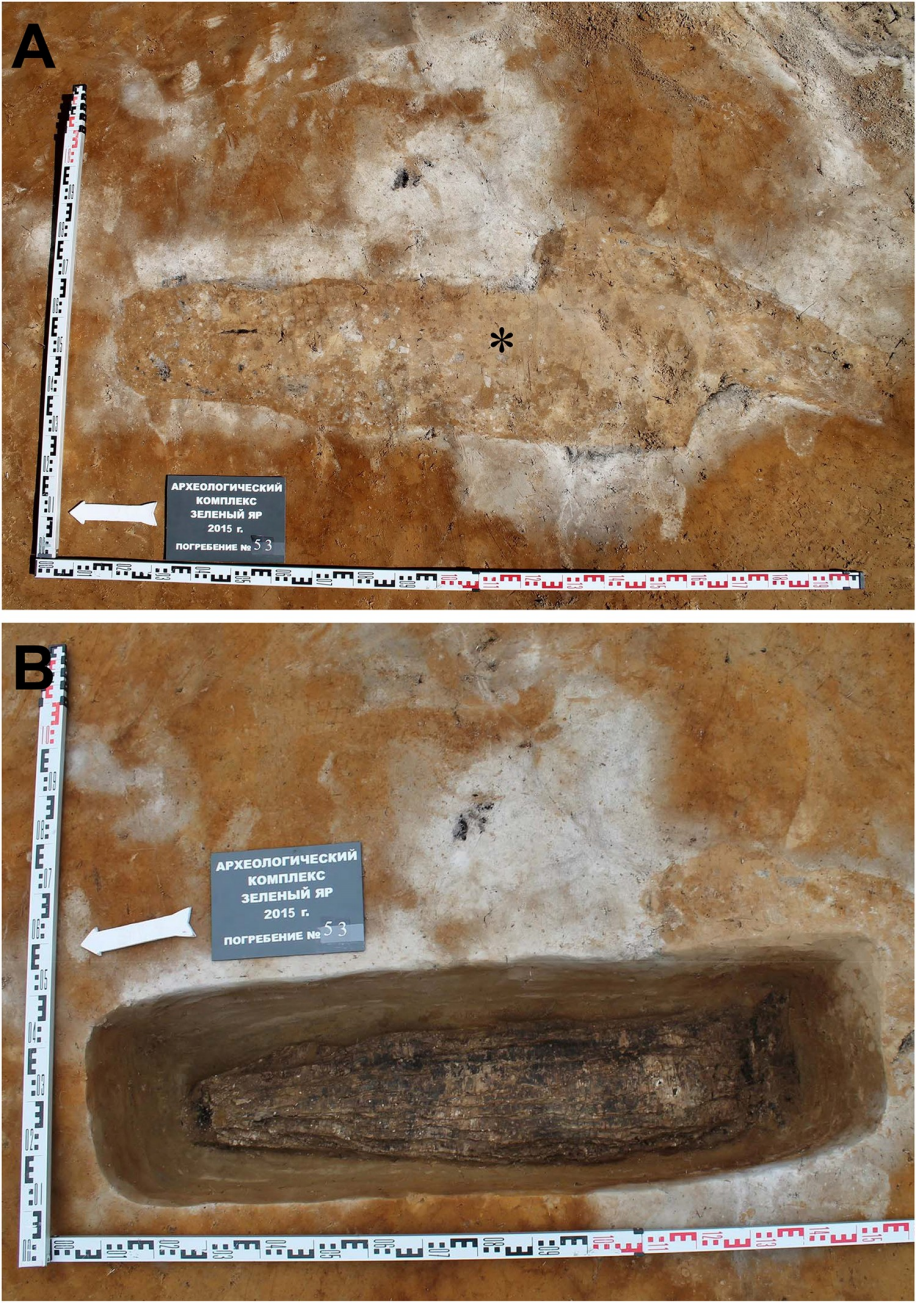
The excavation of burial #53 in the Z-Y grave yard. (A) The shape of the burial (asterisk) was identified on the surface soil. (B) At a depth of -0.35 to -0.45 m, a cocoon of birch-bark overlaps was found.

During the 2013–2016 excavations, a total of 47 burials were newly discovered at the site (Table 1). Burials #55 and #67 were of the paired type. Forty-nine individuals therefore were unearthed. The majority of graves were primary singular burials. Some of the bodies (n = 11) were cocooned in birch bark. The burial practice was evidenced by grave #53, one of the most well-preserved at the Z-Y excavation site. Briefly, the body was oriented along the north-south axis, with a slight declination to the west. The shape of the burial was trapezoidal. Its dimensions were 1.51 m (longitudinal) and 0.36 m (width in the widest part) (Fig 2A). At a depth of -0.35 to -0.45 m, a cocoon made of birch-bark overlaps was found (Fig 2B), inside of which was the body. The head was observed to be protruding at the south end of the cocoon. The dimensions of the cocoon were 1.24 m (longitudinal) by 0.31 m (transverse in the middle part).

**Table 1:** Graves of Zeleny Yar cemetery discovered in 2013–2016.

Burial/Repository Number	Type of Burials	Cocooned	Mummified	Human Remains
36	Primary/Singular			Human teeth only
37	Primary/Singular			Skeleton
38	Primary/Singular			Skeleton; parts of the right and left feet are mummified
39	Primary/Singular			Skeleton
40	Primary/Singular			Skeleton
41	Primary/Singular			No human remains
42	Primary/Singular			Human hair (only)
43	Primary/Singular			Skeleton
44	Primary/Singular			Skeleton
45	Primary/Singular			Human teeth only
46	Primary/Singular			No human remains
47	Primary/Singular			Skeleton
48	Primary/Singular	◯		Skeleton; Both upper limbs of the child, shoulder and forearm are mummified
49	Primary/Singular			Skeleton
50	Primary/Singular			Skeleton
51	Primary/Singular			No human remains
52	Primary/Singular			Human teeth only
53	Primary/Singular	◯	◯	Mummy (young child); 6–7 years old; male
54	Primary/Singular			No human remains
55–1	Primary/Joint			Human teeth only
55–2	Primary/Joint			Skeletons (tibia diaphysis of an adult; a single bone probably from another burial)
56	Primary/Singular			Skeleton
57	Primary/Singular			Skeletons
58	Primary/Singular			Skeletons
59	Primary/Singular			No human remains
60	Primary/Singular			No human remains
61	Primary/Singular	◯		No human remains
62	Primary/Singular			Skeleton
63	Primary/Singular			Human teeth only
64	Primary/Singular	◯		No human remains
65	Primary/Singular	◯		Skeleton
66	Primary/Singular			Skeleton; Disjointed fragments, pelvic region and unidentified soft tissue are mummified
67–1	Primary/Joint		◯	Skeleton; Right forearm with hand and pelvic region are mummified
67–2	Primary/Joint		◯	Skeleton; Part of right arm; shoulder and proximal part of forearm are mummified
68	Primary/Singular		◯	Skeleton; Right upper limb, shoulder, forearm, part of the wrist, right side of the thorax, left pelvic bone with soft tissues are mummified
69	Primary/Singular		◯	Skeleton; Hands and anterior part of abdominal wall are mummified
70	Primary/Singular			Skeleton
71	Primary/Singular			Skeleton
72	Primary/Singular			Skeleton
73	Primary/Singular			Skeleton
74	Primary/Singular			No human remains
75	Primary/Singular		◯	Skeleton; Chest is mummified
76	Primary/Singular	◯		Skeleton
77	Primary/Singular			Skeleton
78	Primary/Singular	◯	◯	Skeleton; Head, pelvic region, left part of the body: lower part of thorax and abdominal cavity, right side of thorax with scapula and shoulder are mummified
79	Primary/Singular	◯	◯	Mummy (adult)
80	Primary/Singular	◯		Skeleton
81	Primary/Singular	◯	◯	Skeletons; mummified pelvis, mummified fragments of hands
82	Primary/Singular	◯	◯	Mummy (baby)

The remains of the graves were moved to the Shemanovsky museum-exhibition complex (Salekhard, Russian Federation). The repository number of specimens is summarized in Table 1. The human samples maintained in the museum-exhibition complex were only allowed to be accessed by relevant researchers. Detailed anthropological examination was performed at the Tyumen Scientific Center of the Siberian Branch of the Russian Academy of Sciences.

**Fig 3:**
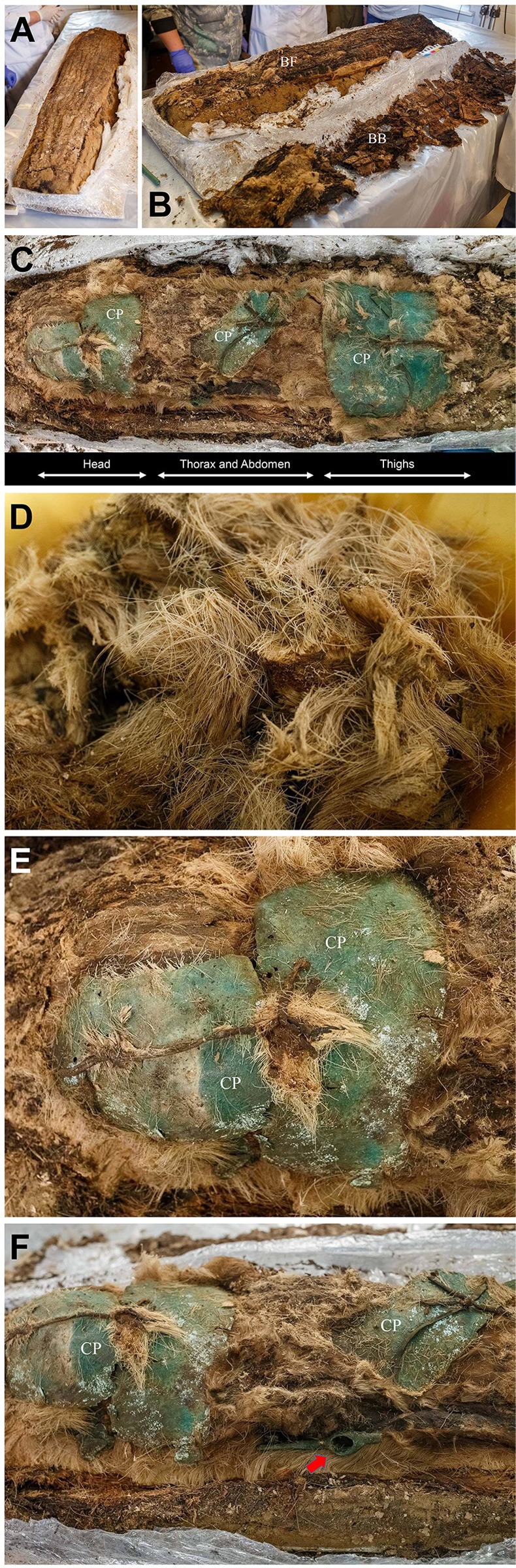
The cocoon structure revealed while dismantling #53 at the lab museum. (A) Multiple birch bark layers of cocoon #53. (B) Bast fiber (BF) was identified after removal of the birch bark surface (BB). (C) Fur veil underneath the bast fiber. Copper plates (CP) were identified above the parts of the face, the thorax, the abdomen, and the thighs. (D) The whole body and all artefacts were covered by a fur veil. (E) Two copper plates (CPs) overlapped in front of the face. (F) The bronze battle axe (indicated by arrow).

Cocoon #53, upon dismantling in the lab, was found to be composed of multiple layers of birch bark (Fig 3A). Longitudinal creases were identified on the birch-bark surface and also on the strong woody plant fiber (bast fiber) found beneath it (Fig 3B). The creases seem to have been made artificially and intentionally, possibly trimmed by human teeth. Underneath the bast fiber, we discovered a fur veil covering the cultural and human remains (Fig 3C and 3D). Various artefacts such as leather, bronze and iron objects were found between each fur veil layer (Fig 3C, 3E and 3F). Two copper plates overlapped in front of the face. Bronze items also were retrieved from both sides of the head. Copper plates had been placed diagonally upon the mummy’s thorax and abdomen (from upper right to lower). In the same place were a bronze battle axe (length: 11 cm), a bronze zoomorphic artefact, and a broken iron object. The blade of the axe was oriented towards the head of the body (Fig 3F). The thighs were covered with two rectangular overlapping copper plates, the distal over the proximal one (Fig 3C).

Upon removal of the fur veil from specimen #53, the well-preserved human remains were exposed. The anthropologist on our team estimated this case to be a boy of six to seven years of age. Each step of the procedure was photographed and videotaped. The artefacts (birch bark, bast and wood) were treated in the restoration workshop of the museum immediately after their retrieval.

## Materials and methods

### Mummies

In the course of our excavations of 2013–2016, we examined the mummies found inside Z-Y graves #53, #67–1, #67–2, and #68. The mummies’ archaeological and anthropological information is summarized in Table 2. The scientific techniques used in this study are indicated therein as well.

**Table 2:** Specimens of Z-Y mummies examined in this study.

Burial No.	Sex	Age	Samples	CT Radiology	Histology: LM	Histology: TEM	aDNA analysis
53	M	6–7	Whole Body	◯			
			Liver		◯		
			Brain				◯
			Hair			◯	
67–1	M	Adult	Small Intestine		◯	◯	
			Urinary Bladder		◯	◯	
			Inferior Vena Cava				◯
			Peritoneum and muscles				◯
			Urinary bladder				◯
			Intestine			◯	◯
			Rectum			◯	◯
			Abdominal aorta				◯
			Cecum/appendix				◯
			Skin/Muscle				◯
67–2	M	20–25	Forearm				◯
68	M	Adult	Muscle (Gluteus)		◯	◯	
			Lung and Pleura		◯	◯	◯
			Skin		◯	◯	◯

### Gross examination and autopsy

With the naked eye, we examined the mummified bodies for any pathological signs. Since some parts of the skin and muscle had already decomposed (#53, #67–2, and #68), their internal organs could be examined and sampled without the necessity of invasive procedures. Meanwhile, an autopsy was performed in only one case, #67–1 (abdominal and pelvic regions), taking care to minimize any damage to the body.

### CT radiography

For specimen #53, in order to maintain the original form of the cocoon, CT radiographs were used to estimate the situation inside the birch bark prior to the investigation. The Brilliance 16 Philips CT scanner at the City Clinical Hospital of Salekhard, Russia, was used for multi-slice CT scanning. Three cycles of scanning were performed on the mummy using between-slice intervals of 1.5 and 2.0 mm. The scanner parameters were as follows: X-ray tube MRC, 8.0 MHU; heat capacity of tube, 8 MHU; generator, 60 kW; configurations of slice thicknesses, 16 × 0.75 mm, 16 × 1.5, 8 × 3, 4 × 4.5, 2 × 0.6 mm; resolution, 24 pairs of lines/cm; rotation time, 0.5 sec (0.4 sec optional); reconstruction speed, 6 images/sec. The reconstructed axial images were transferred to a workstation for post-processing. Using these, coronal and sagittal multi-planar reformat (MPR) and volume rendering (VR) images were also obtained. The CT study allowed us to prepare a 3D model of the skull to thoroughly describe the skeleton and the remaining soft tissues.

### Histology

Using both light and electron microscopes, histological examinations were performed on mummy samples from the small intestine (#67–1), urinary bladder (#67–1), gluteus muscle (#68), skin (#68) and hair (#53). For light microscopy, the samples were rehydrated in Ruffer’s solution (5:3:2 for distilled water: absolute ethanol: 5% sodium carbonate) [13]. After fixation by 4% paraformaldehyde, the samples were treated with an increasing series of sucrose (10–30% w/v). The total period for rehydration, fixation and sucrose treatment took two weeks. Tissues were then embedded in an optimal cutting temperature (OCT) compound and frozen rapidly in 2-methylbutane pre-cooled to its freezing point with liquid nitrogen. Tissue specimens were cut into 12–20 um sections on the cryostat, then were thaw-mounted on gelatin-coated microscopic slides. They were finally stored at 20°C until required for experiments. Sections were stained using the hematoxylin-eosin and Masson’s trichrome stain, following the methods described by Sheehan and Hrapchak [14].

Observation via scanning electron microscope (SEM) or transmission electron microscope (TEM) followed the methods of Hayat [15] and Bozzola & Russell [16]. Briefly, the sample was pre-fixed by immersion in 2% paraformaldehyde– 2.5% glutaraldehyde in neutral 0.1 M phosphate buffer, and post-fixed for 2 h in 1% (w/v) osmic acid dissolved in phosphate-buffered saline (PBS). As for the SEM study, the sample was next treated in a graded ethanol series and isoamyl acetate and dried in a critical point dryer (HCP-2, Hitachi, Japan). Pt-Pd coating was performed using an ion sputter (E-1030, Hitachi, Japan). The sample was finally observed using an S-4700 SEM (Hitachi, Japan). For the TEM study, after post-fixation, the sample was dehydrated in graded ethanol and embedded in Epon812 (EMS, Fort Washington, PA, USA). After ultrathin sections were cut and mounted on nickel grids, coating with Formvar film and uranyl-lead counter-staining were performed. The sections were finally observed under an H-7600 TEM (Hitachi, Japan).

### Ancient DNA analysis

aDNA molecules readily degrade into very short fragments and are often detected at very low concentrations [17, 18]. This is quite different from modern DNA. Because of the possibility of contamination with modern DNA during analysis [19], strict laboratory procedures must be followed [18, 20], which was the case in our study [20].

To meet the criteria for minimization of modern DNA contamination, every research participant wore sterilized gowns, head caps, gloves, and masks. Every tool used in this study was sterilized before use. The obtained samples were stored in sterilized containers. Nobody was permitted to have contact with the samples without permission.

The bone samples’ (#34 and #67–1) surfaces were abraded and exposed to UV for 20 min. Brain (#53), aorta (#67–2) and pleura (#68) samples were also exposed to UV for 20 min before use. The bone samples were treated with 5.4% sodium hypochlorite; they were then powdered by SPEX 6750 Freezer ⁄ Mill (SPEX SamplePrep, Metuchen, USA). All of the samples (0.5g) were incubated in 10 ml of lysis buffer (pH 8.0; including 50 mM of EDTA; 1 mg/ml of proteinase K; 1% SDS; 0.1M DTT) at 56°C for 48 hrs. Total DNA was extracted with an equal volume of phenol/chloroform/isoamyl alcohol (25:24:1). Extracted DNA was purified with the QIAmp PCR purification kit (QIAGEN, Hilden, Germany).

The extracted DNA (10 μl) was quantified using a NanoDrop ND-1000 Spectrophotometer (Thermo Fisher Scientific, MA, USA), and treated with 1 unit of uracil-DNA-glycosylase (New England Biolabs, USA) for 30 min at 37°C. Next, aDNA (40 ng) was mixed with the reagent premix containing 1X AmpliTaq Gold 360 Master Mix (Life Technologies, USA) and 10 pmol of each pre-designed primer set (Integrated DNA Technology, Iowa city, USA). The PCR conditions applied in this study were as follows: pre-denaturation at 95°C for 10 min; 42 cycles of denaturation at 95°C for 30 secs; annealing at 56°C for 30 secs; extension at 72°C for 30 secs; final extension at 72°C for 10 min. PCR amplification was performed using a SimpliAmp Thermal Cycler (Applied Biosystems, Waltham, USA). The information on the primer sets is summarized in S1 Table.

The PCR products were separated on 2.5% agarose gel (Invitrogen, Carlsbad, USA) and then stained with ethidium bromide. They were photographed using a Vilber Lourmat ETX-20.M equipped with Biocapt software (Vilber Lourmat, Collégien, France). The PCR amplicons were isolated using a QIAquick Gel Extraction Kit (Qiagen, Hilden, Germany). Bacterial transformation was performed with the pGEM-T Easy Vector system (Promega Corporation, Madison, USA). Transformed bacteria were grown on an agar plate containing ampicillin (50 μg/ml), 0.5 mM IPTG, and X-GAL (40 μg/μl) for the next 14 hrs. After selected colonies were grown once again in LB media for 12 hrs, the purification of cultured bacteria was performed using a QIAprep Spin Miniprep kit (Qiagen, Hilden, Germany).

Sequencing of each strand was performed using an ABI Prism BigDye Terminator v3.1 Cycle Sequencing Ready Reaction Kit (Applied Biosystems, Waltham, USA) on the 3730xl Automated Sequencer (Applied Biosystems, Waltham, CA). The obtained DNA sequences were aligned by MEGA7 [21]. The consensus sequences were compared with the revised Cambridge Reference Sequence (rCRS; accession number: NC_012920) in order to identify the sequence differences. Haplogroups of the mtDNA control region were assigned by using *MitoTool* (http://mitotool.org; version Phylotree 16 rCRS) [22] and *Jameslick* (https://dna.jameslick.com; Data Version 17.0) [23]. As for our histological and aDNA analyses, the Institutional Review Board (IRB) of Seoul National University Hospital confirmed that they could be exempt from board review (IRB No. 2017–001). Our study was conducted in accordance with the Vermillion Accord on Human Remains, World Archaeological Congress.

## Results

### Gross examination

**Fig 4:**
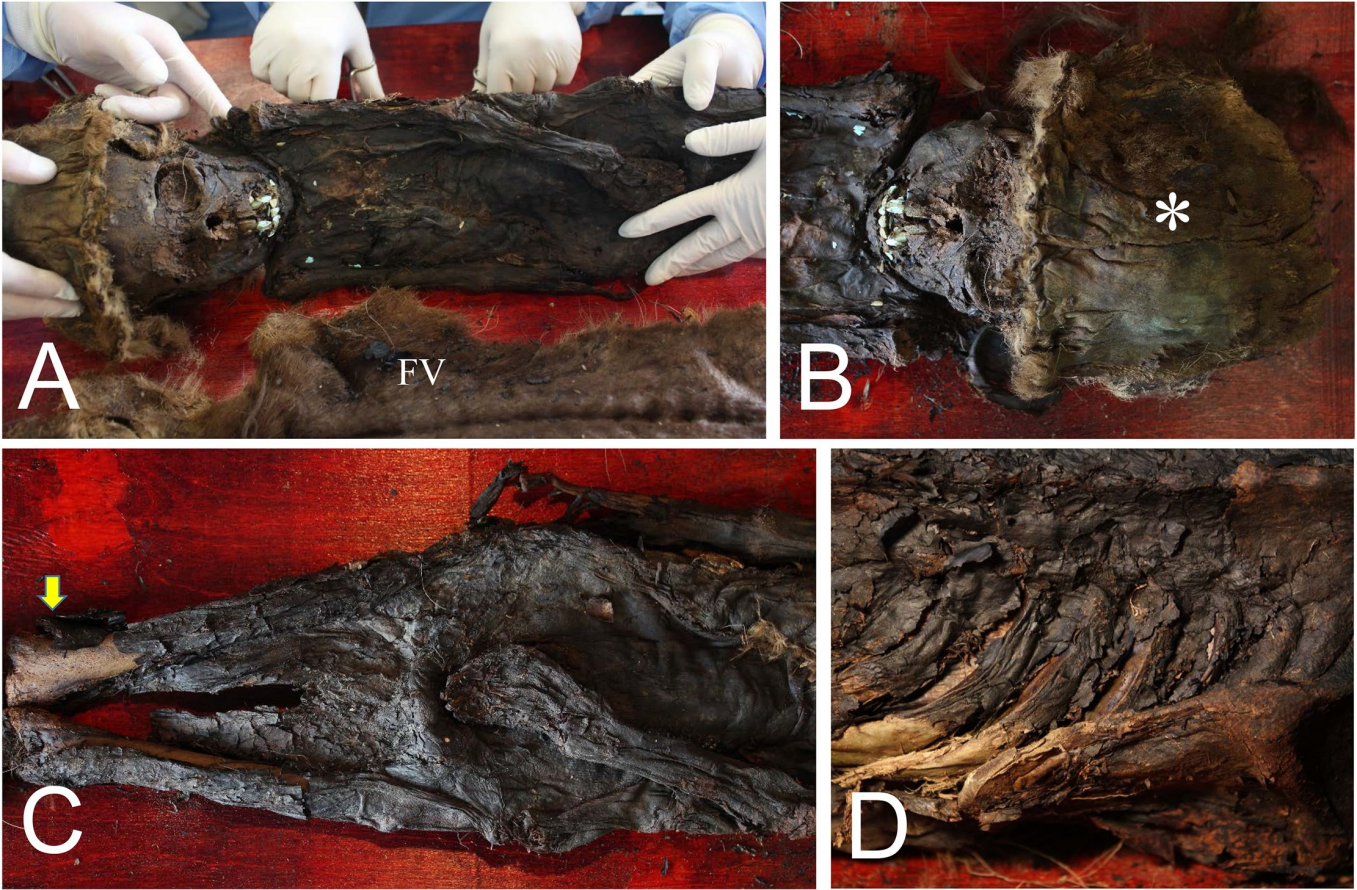
Gross examination of #53. (A) The whole body of #53 exposed after the removal of the fur veil (FV). (B) The cap (asterisk). (C) The mummified body. Skeletonized part of lower extremity is indicated by an arrow. (D) Ribs and intercostal spaces can be visible on the back of the mummy.

In this study, due to the relatively well-preserved conditions, gross examination was mainly concentrated on the #53 (child) mummy. The whole mummified body was exposed after removal of the fur veil (Fig 4A). The mummy wore a cap (Fig 4B). The mummified body lay on his back, with the legs straight. The face, the upper limbs, the chest, the abdomen and the thighs were completely mummified, though part of the lower extremities was skeletonized (Fig 4C). Specimens inside the thoracic cavity were collected from defects in the intercostal space (Fig 4D). We could not find any specific pathological alterations on the skins of any of the Z-Y mummies.

### CT radiography

**Fig 5:**
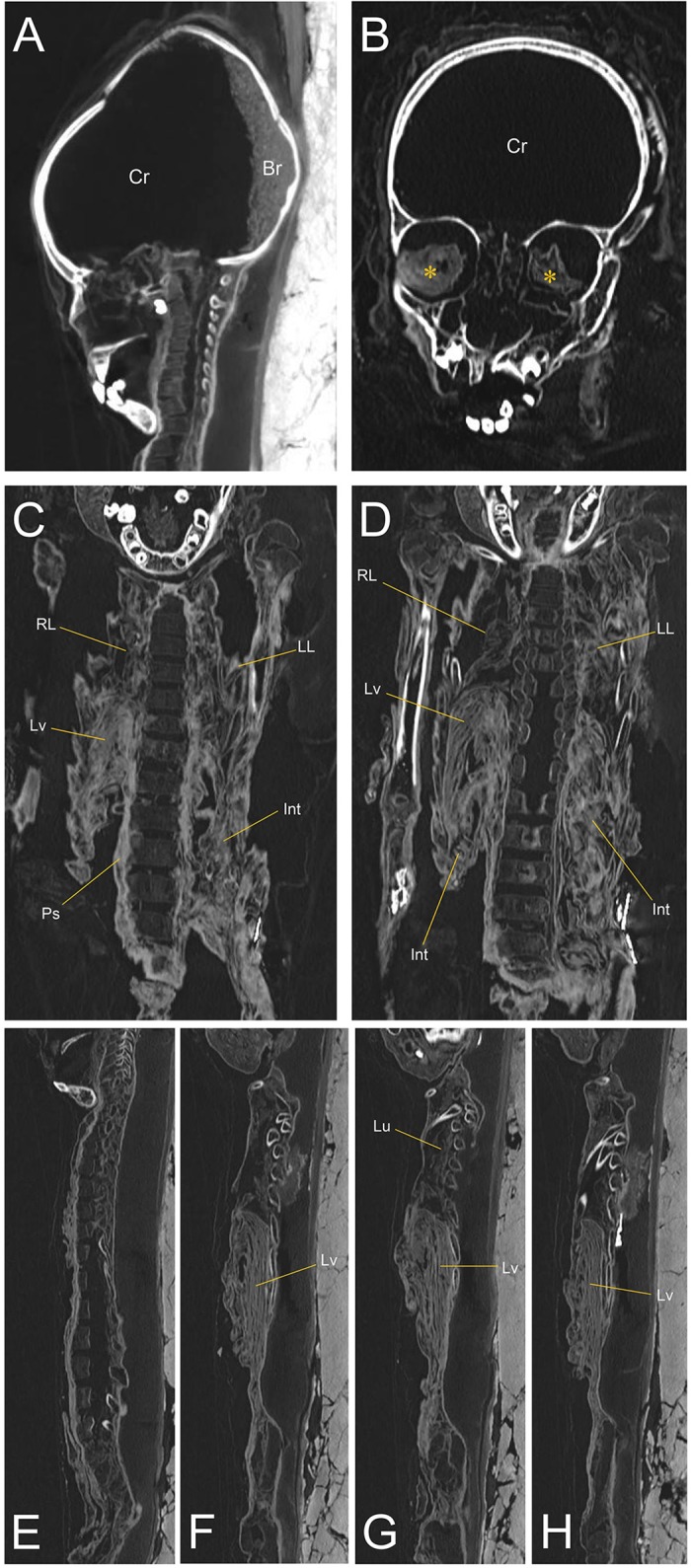
CT images of the child mummy #53. (A) Sagittal and (B) coronal views of the head CT images. Br, brain remnant. Cr, cranial cavity. Asterisks indicate the eyeballs. (C) and (D) Coronal views of trunk. RL, right lung; LL, left lung; Lv, liver; Int, intestine; Ps, psoas muscle. (E) to (H) Sagittal views of trunk. Lu, lung.

To view the remnant mummified organs, CT radiography was performed on mummy #53. The CT analysis showed that the internal organs were still preserved well inside the body cavities. We identified the brain remains in the cranial cavity (Fig 5A and 5B). Eyeball soft tissues were found within both orbits (Fig 5B). The mummified organs (lung, liver, abdominal intestine, muscle beside the vertebral column, etc.) were identified in the thoracic and abdominal cavities (Fig 5C and 5D). On coronal and sagittal CT images, we also found the lungs, liver and intestines (Fig 5E–5H). The mummified internal organs were shrunken toward the back, almost certainly as a result of the action of gravity over the long-time course (Fig 5A–5H). We carefully sampled the specimens by the guidance of CT images.

### Autopsy

**Fig 6:**
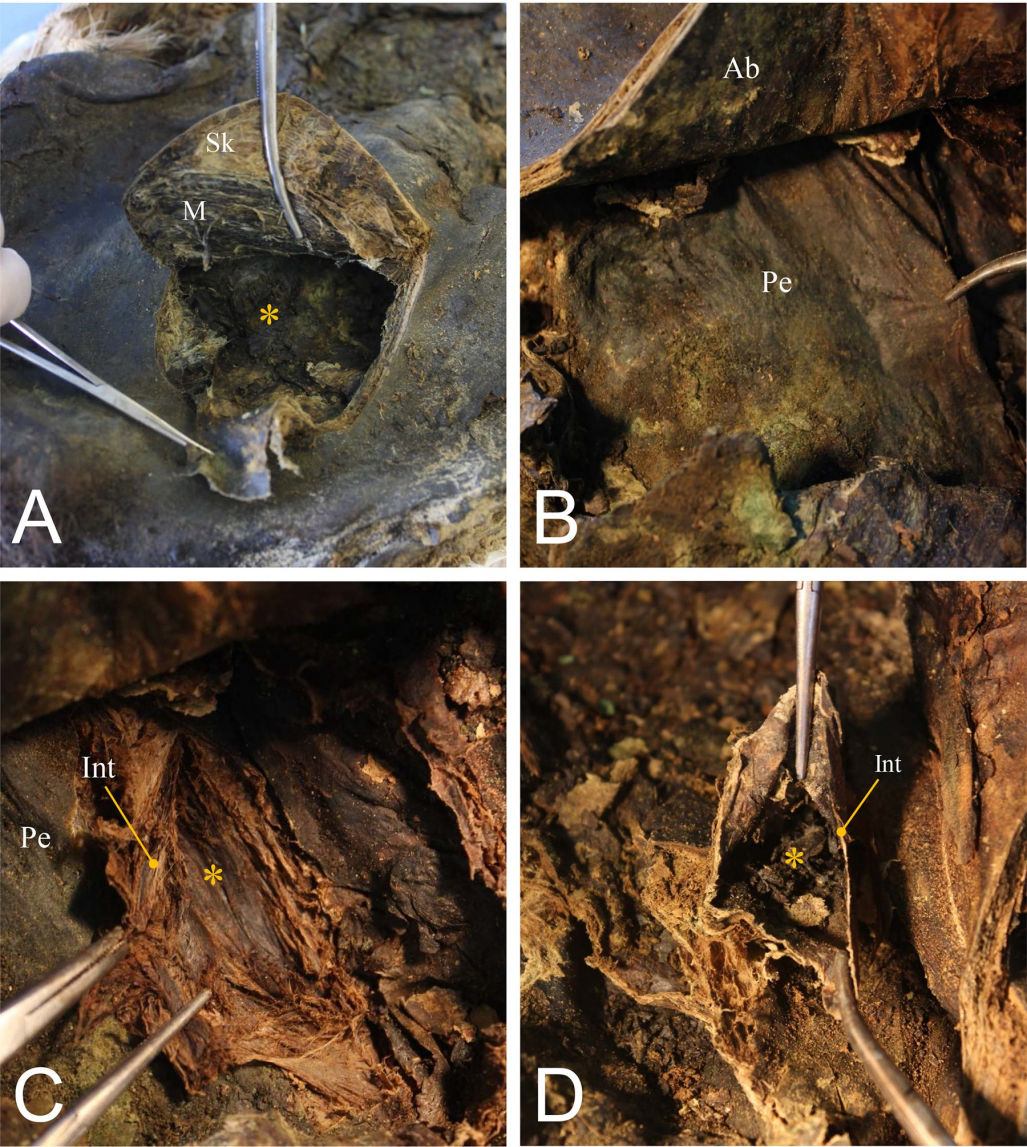
Autopsy of #67–1. (A) Incision of body wall. Sk, skin; M, muscle; internal organs marked by asterisk. (B) Peritoneum (Pe). Ab, abdominal wall. (C) Incision made on peritoneum and intestines (Int). Aorta (asterisk) could be exposed. (D) Note very thin intestinal wall (Int). Coprolite (asterisk) filling inside the lumen.

In the autopsy of #67–1, we incised the skin to expose the mummified organs of the abdominal and pelvic cavities (Fig 6A). The peritoneum could be seen inside the body cavity. The intestines were well preserved (Fig 6B and 6C). We found coprolites filling the intestinal lumen. The intestinal wall had become very thin (Fig 6D). Specimens were collected during the autopsy.

### Histology

**Fig 7:**
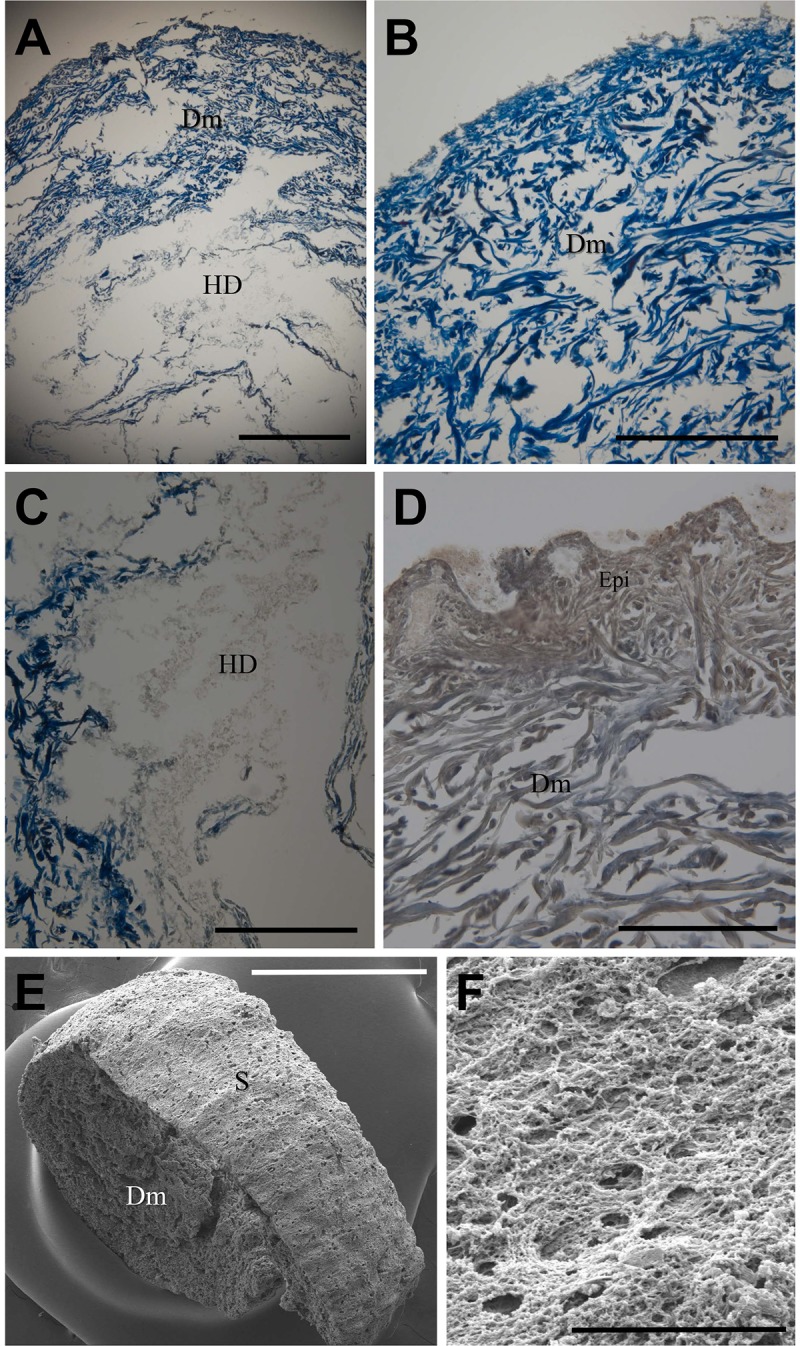
Histology of Z-Y mummies. Skin of #68. (A) Dermis (Dm) and hypodermis (HD). Scale bar, 1mm. (B) Dermis mainly composed of collagen fibers. Masson’s trichrome staining. (C) Tissue debris in HD. (D) Epithelial cell debris in skin specimen. Scale bars in (B) to (D), 500 um. (E) and (F) SEM images. S, surface of skin. (F) Collagen fibers in dermis. Scale bars in (E), 1 mm; in (F), 100 nm.

Under light and electron microscopy, we observed the histological morphologies of the small intestine (#67–1), urinary bladder (#67–1), gluteus muscle (#68), skin (#68) and hair (#53). As for the skin (#68), the distinction between the dermis and hypodermis was clear (Fig 7A). The dermis was mainly composed of collagen fibers stained by Masson’s trichrome (Fig 7B). Seriously degraded tissue debris could be observed in the hypodermis (Fig 7C). Epithelial remnants were often found in some parts of the skin epidermis (e.g. of gluteal region) (Fig 7D). Also, the presence of collagen fibers in the dermis was confirmed on SEM images (Fig 7E and 7F).

**Fig 8:**
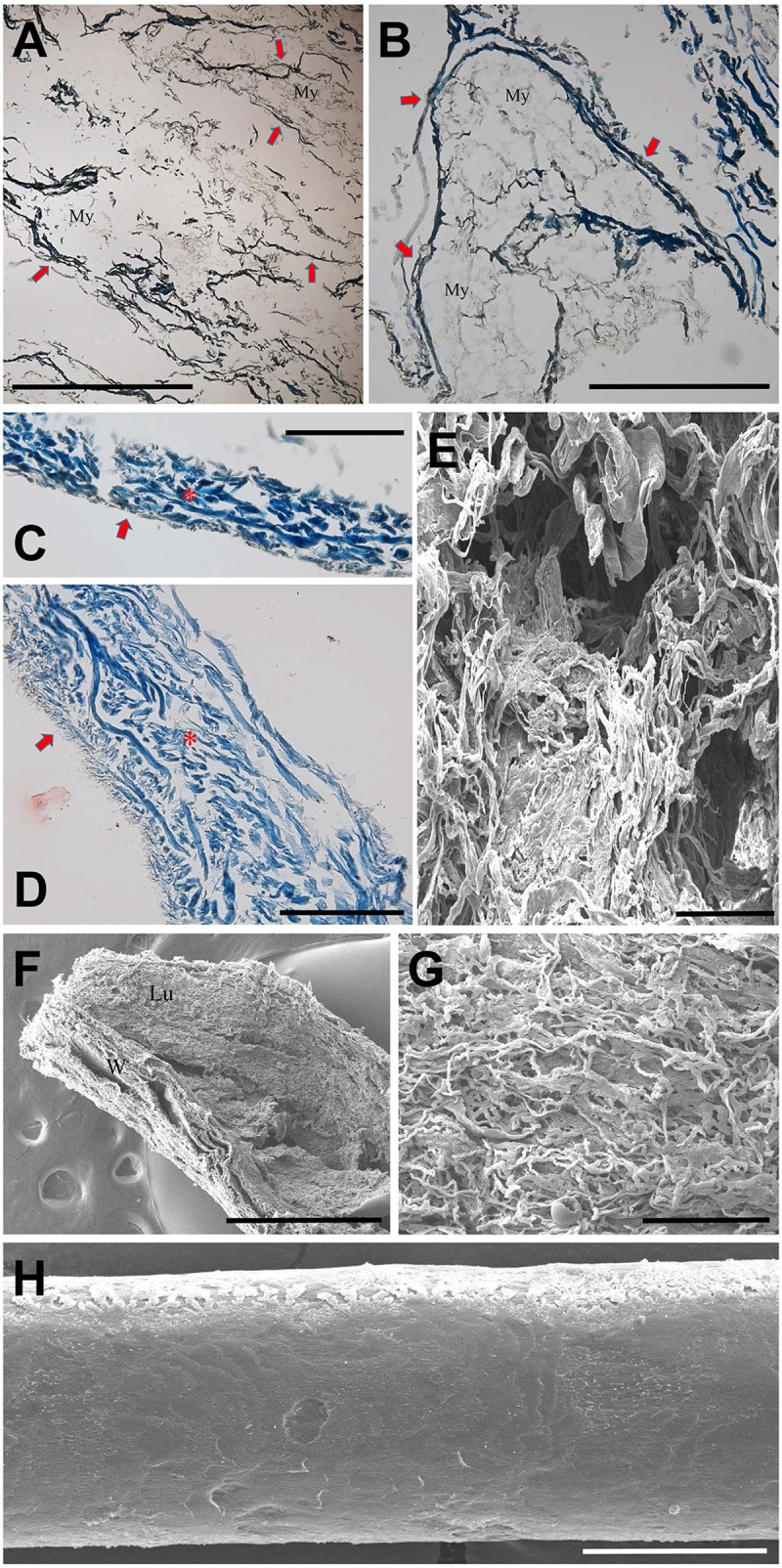
Histology of Z-Y mummies’ muscle, urinary bladder, small intestine and hair. (A) and (B) Transverse sections of gluteus muscle #68. Collagen remnants of perimysium (indicated by arrows). Myocytes (My) disappeared. Scale bars, 500 um. (C) to (E) Urinary bladder of #67–1. (C) and (D) Atrophic transitional epithelium (indicated by arrows). Collagen fibers remnants of bladder wall (asterisks). Scale bar, 200 um. (E) SEM of urinary bladder. Note collagen fibers. Scale bar, 100nm. (F) and (G) Small intestine of #67–1. (F) Lu, luminal surface of intestine. W, intestinal wall. Scale bar, 1mm. (G) Collagen fibers in the wall of small intestine. Scale bar, 100 nm. (H) Ultramicroscopic image of hair collected from #53. Scale bar, 50 nm.

We then observed histological findings of gluteus muscle (#68; Fig 8A and 8B) and urinary bladder (#67–1; Fig 8C–8E). Briefly, collagen remnants of perimysium were seen in the muscle transverse section, but the myocytes had already disappeared (Fig 8A and 8B). In the case of the urinary bladder, the transitional epithelium had become atrophic, thus leaving only very thin film-like linings (Fig 8C and 8D). Submucosal cells and myocytes were not observed in the bladder wall, though collagen fibers still remained (Fig 8E). The mummified small intestine also was mainly composed of collagen fibers (Fig 8F and 8G). Fig 8H is an ultramicroscopic image of hair collected from #53. Although the appearance of the mummified hair looked fine by the naked eye, in the SEM image, the hair surface showed serious degeneration on the scale edges.

### Ancient DNA analysis

**Fig 9:**
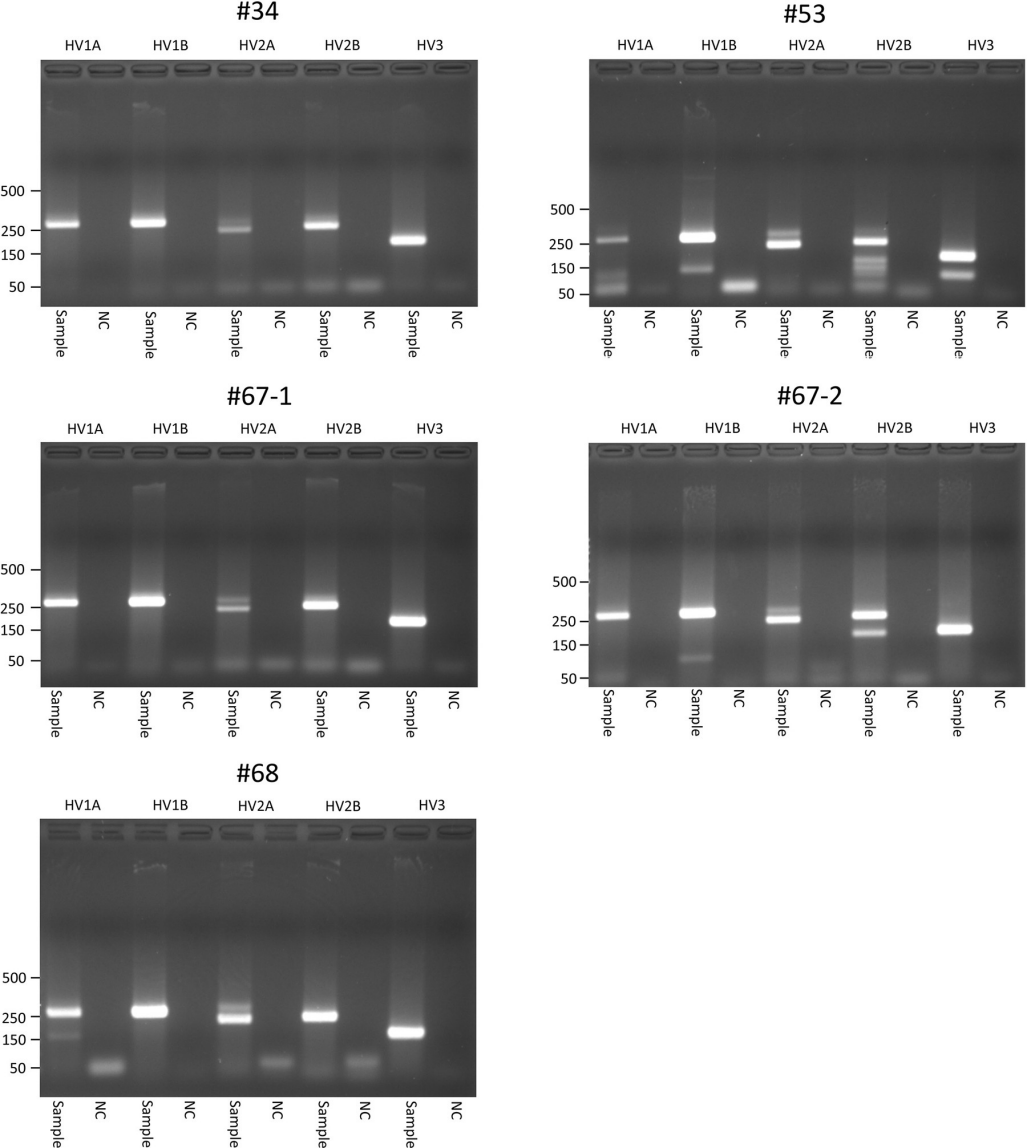
Agarose gel electrophoresis for the amplicons of the mtDNA D-loop region of Z-Y mummies. Negative controls (extraction controls), NC. Specific bands for HV1A, 267bp; HV1B, 267bp; HV2A, 226bp; HV2B, 235bp; HV3, 167bp.

**Fig 10:**
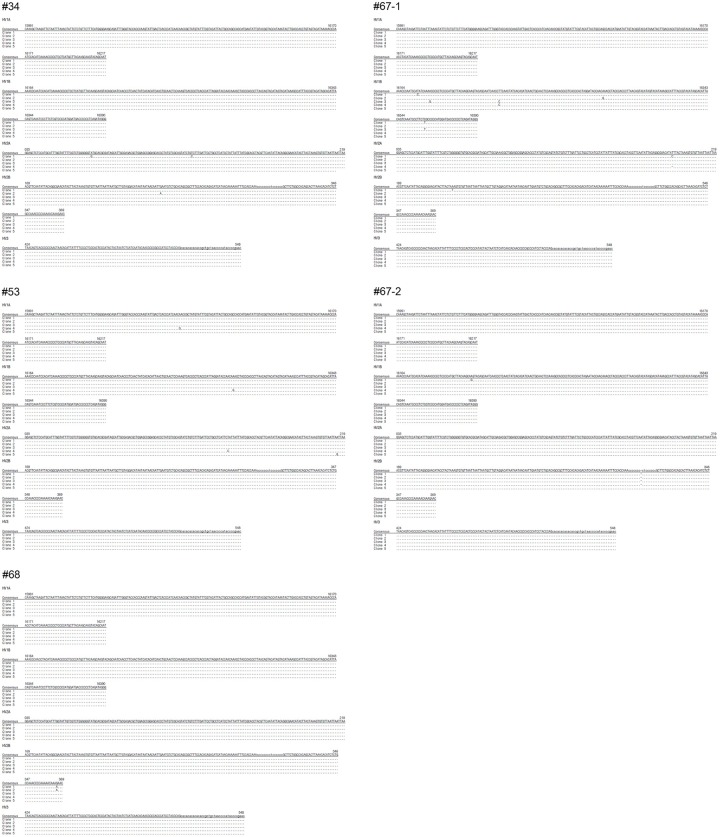
Cloned sequences of mtDNA D-loop region of Z-Y mummies. The consensus sequence could be obtained by the cloned sequences.

In our agarose gel electrophoresis, the PCR-amplified products showed specific bands for the amplicons of the mtDNA D-loop region of the Z-Y mummies, while the negative controls (extraction controls) did not exhibit any amplified bands (Fig 9). Most cloned sequences were identical except for some clones showing single nucleotide substitutions (Fig 10). Consensus sequences of the mtDNA D-loop were obtained from the clone sequences for each Z-Y mummy sample (Fig 10). The consensus mtDNA mitotypes are summarized in Table 3. The authenticity of aDNA analysis could be confirmed by comparison of mtDNA haplotypes between the Z-Y mummies and a participating researcher (Table 3). Since we did not find any identical sequence between them, the DNA obtained from the Z-Y mummies was highly likely to be endogenous aDNA but not the outcome of modern DNA contamination. The Z-Y mummies’ mtDNA haplotypes were determined to belong to five different haplogroups: U5a (#34), H3ao (#53), D (#67–1), U4b1b1 (#67–2), and D4j8 (#68) (Table 3).

**Table 3:** Compared sequencing analysis result of mtDNA hypervariable region.

Sample	Hypervariable region			Haplogroup
	HV1(15991–16390)	HV2(034–369)	HV3(423–548)	
#34	16192T, 16256T, 16270T	73G, 131C, 263G, 309.1C, 315.1C	rCRS	U5a
#53	16239T, 16256T, 16270T	263G, 315.1C	rCRS	H3ao
#67–1	16176C, 16223T, 16362C	73G, 94A, 194T, 263G, 309.1C, 315.1C	489C	D
#67–2	16274A, 16311C, 16356C	73G, 146C, 152C, 195C, 263G, 309.1C, 315.1C	499A, 513.1CA	U4b1b1
#68	16172C, 16174T, 16223T, 16362C	73G, 263G, 309.1C, 315.1C	489C	D4j8
Researcher 1	16093C, 16176T, 16223T, 16362C,	73G, 94A, 194T, 263G, 309.1C, 315.1C	rCRS	D4e1a1

## Discussion

### Arctic mummies reported to date

Relatively few anthropological examinations have been reported on permafrost mummies, especially those from arctic or subarctic regions. Some of the rare mummies found have been those of the Inuit, the people living and prospering in Alaska and Greenland prior to European colonization [24,25]. For examples, the frozen body of a native woman (dated 200–500 CE) was found on Saint Lawrence Island, Alaska [5,24]. In 1982, the remains of five native people were discovered in a crushed house at Utqiagvik, the northernmost point of Alaska. In 1994, the frozen body of a young girl from the semi-nomadic Thule culture (800–1200 CE) was also discovered at Ukkuqs, Alaska [5]. Studies on arctic mummies discovered in Greenland have been conducted on a much larger scale. In the Pisisarfik Mountains of the Nuuk/Godthaab district, 16th century mummies (n = 6) wearing well preserved garments were found [24,25]. Eight 15th century mummified bodies (six adults, two children) were found in a rock crevice at Qilakitsoq, Northwestern Greenland. The Qilakitsoq case, still one of the largest single mummy finds in an arctic region, offers unique insight into the Thule culture [1,24,25].

Thus far, the discussion has concentrated on Western-hemispheric arctic countries (Canada, the U.S. [Alaska], Greenland). However, significant permafrost-mummy finds have also been made in the Eastern hemisphere, specifically the Russian Federation. Briefly, a great number of skeletons and mummies (n = 140) in 16^th^ to 19^th^ century tombs have been discovered in Eastern Siberia, specifically Central Yakutia, the Vilyuy River basin, and the Verhoyansk area of the Sakha Republic (Yakutiya). Most of the bodies were frozen due to inhumation in permafrost. This find may represent the largest ever number of frozen mummies for any single study or series of studies in the world. The bodies, moreover, were found in an exceptional state of preservation [7–9].

As for the Western Siberian arctic, as noted earlier, pioneering scholars conducted studies on Z-Y mummies between 1999 and 2002 [10–12]. Despite the invaluable data obtained from those studies, gaps in the overall understanding of the Z-Y mummies remain. In this regard, our present research on Z-Y mummies newly discovered during a 2013–2016 expedition is very significant to concerned researchers. Using systematically integrated techniques of gross morphology, radiology, autopsy, histology, and aDNA analysis, we have sought to unveil the biomedical aspects of those ancient native Western Siberian people.

### Preservation status of Z-Y mummies

Natural mummification in an arctic permafrost region ensures excellent preservation status, due possibly to favorable local conditions with year-round low temperatures and low air humidity [1,24]. Correspondingly, the internal organs of the Z-Y mummies were well preserved, as evidenced in the current study. As the bodies had not been treated by any artificial preservatives, and as the internal organs had not been removed during embalmment, the academic value of the Z-Y mummies was deemed to be very high indeed.

We noted that the preservation pattern of the Z-Y mummies, as revealed by our gross, radiological, and histological analyses, was somewhat similar to that obtained from other natural mummification cases. First, via autopsy, we observed that various internal organs remained, albeit seriously dehydrated, deformed and displaced. In the radiological study on Z-Y mummy #53, the internal organs had collapsed to the dorsal side of the body cavity. The most prominent organs observed were the brain, lung and liver. Interestingly, in the cases of 16^th^ to 18^th^ century Korean cadavers that had been naturally mummified, the internal organs were distorted, shrunken and dorsally displaced on CT images as well, almost certainly by the long-term actions of dehydration and gravity [26]. This fact can be considered to be evidence suggesting that the preservation pattern of the Z-Y mummies is similar to those of at least some other natural mummies, and, therefore, that the radiological knowledge gained via examination of the latter (e.g. Korean mummies) could be applied to the CT interpretation of the Z-Y mummies.

As for the microscopic morphology, the Z-Y mummy tissues were preserved in a very unique pattern. The findings were as follows: change of skin histology (i.e. remarkable atrophy of the epidermis but maintained dermis thickness); disappearance of cells or other organic materials, and abundance of connective tissues (e.g. collagen fibers). These findings are very similar to the histological results reported for other natural mummies. In the cases of the 5,200-year-old Tyrolean Ice Man and Joseon-period Korean mummies, unique microscopic patterns pertinent to the state of preservation of the respective mummified structures were observed. The skeletal and connective tissues were generally intact, whereas the epithelial as well as blood cells had almost entirely disintegrated [27,28].

In sum, the radiological, macroscopic and microscopic similarities often seen between the Z-Y and other natural mummies demonstrate that the former share many anatomico-histological traits with the latter, though the respective mummification processes occurred in very different and unique environments.

### Ancient DNA analysis

aDNA analysis has become a well-established technique for investigation of the genetic affiliations of ancient samples obtained at archaeological sites, leading either to revelation of their biological origins or elucidation of their relationships with descendants [29,30]. The mummies discovered at Qilakitsoq in Northwestern Greenland provide good examples of aDNA analysis. In that study, ancient DNA analysis was performed on hair and nail specimens. The data indicated probable family relationships between and among at least some of the mummified individuals, though those genetic ties proved more complex than originally postulated [25]. As for the human remains excavated at two archeological sites in Western Siberia, Sato [12] determined that their mtDNA haplogroups could be assigned to B4, C4, G2, H, and U.

In our study on the Z-Y mummies excavated in 2013–2016, every haplogroup that we identified (#53, H3ao; #67–1, D; #67–2, U4b1b1; #68, D4j8) is commonly found among Siberian native people. For instance, U4 (#67–2) is one of the most frequently observed haplogroups (16.3%) among the native population of Western Siberia [12,31,32]. Haplogroup U5, though of a somewhat lower frequency (8.8%), is representative of both Western and Central Siberia [33].

On the other hand, haplogroups D (#67–1 and #68) and H (#53) are commonly seen in East Eurasia as well as Iberia and surrounding areas, respectively [34,35]. D4 is the highest-frequency haplogroup in the Korean mtDNA pool (23.8%), and is widespread in Northeast Asia and especially among Japanese (36.9%), Korean-Chinese (21.6%), and Manchurians (20.0%) [12,35]. Although the frequency peaks of haplogroups D and H are centered in regions outside of Western Siberia [12,34,35], they have been reported from modern Western Siberian populations as well, such as the Khanty (21.3%) and Mansi (8.2%) for haplogroup D [12] and the Mansi of the Lower Ob River basin (14.3%) for haplogroup H [31]. Haplogroup D4j (#68) might have appeared during the Siberian Neolithic period [36], and is still detected in a variety of Southern Siberian populations [37].

Meanwhile, haplogroups A, J, and C4, which are commonly observed among the Western Siberian native population [12,31,32,38], were not identified in the current study on Z-Y mummies. Overall, our aDNA analysis demonstrated that the Z-Y mummies are a unique combination of Western (H3, U4 and U5)- and Eastern Siberia-specific (D and D4) mtDNA haplogroups.

## Conclusion

At the Z-Y burial ground in the Western Siberian arctic, 12^th^ to 13^th^ century permafrost mummies were unearthed. After removal of the multi-layered birch-bark cocoon from each specimen, interdisciplinary studies were performed using various scientific techniques. From our gross anatomical, histological, radiological and molecular-biological analyses, we were able to secure invaluable original data on historical native Siberian populations. This information will form the foundation of successful future studies on medieval mummies discovered in the Western Siberian arctic.

## Supporting information

S1 Table Primer sets used in this study. (DOCX)
