# Supplementary material for: Medieval mummies of Zeleny Yar burial ground in the Arctic Zone of Western Siberia
Source: PLoS One. 2019 Jan 25;14(1):e0210718. doi: 10.1371/journal.pone.0210718 (PMC6347368; doi:10.1371/journal.pone.0210718)
Supplement: S1 Table — (DOCX) [file pone.0210718.s001.docx]

S1 Data.

Primer sets used in this study

| Region | Set | Primer | 5’ to 3’ | Length  (bp) |
| --- | --- | --- | --- | --- |
| HVI  (15991-16390) | HV1A | F15971 | TTA ACT CCA CCA TTA GCA CC | 267 |
|  |  | R16237 | TGT GTG ATA GTT GAG GGT TG |  |
|  | HV1B | F16144 | TGA CCA CCT GTA GTA CAT AA | 267 |
|  |  | R16410 | GAG GAT GGT GGT CAA GGG AC |  |
| HVII  (034-369) | HV2A | F015 | CAC CCT ATT AAC CAC TCA CG | 226 |
|  |  | R240 | TAT TAT TAT GTC CTA CAA GCA |  |
|  | HV2B | F155 | CTA TTA TTT ATC GCA CCT | 235 |
|  |  | R389 | CTG GTT AGG CTG GTG TTA GG |  |
| HVIII  (423-548) | HV3 | F403 | TCT TTT GGC GGT ATG CAC TTT | 167 |
|  |  | R569 | GGT GTA TTT GGG GTT TGG TTG |  |
